# Stability from Structure: Metabolic Networks Are Unlike Other Biological Networks

**DOI:** 10.1155/2009/630695

**Published:** 2008-12-17

**Authors:** P van Nes, D Bellomo, MJT Reinders, D de Ridder

**Affiliations:** 1 Information and Communication Theory Group, Faculty of Electrical Engineering, Mathematics and Computer Science, Delft University of Technology, Mekelweg 4, 2628 CD Delft, The Netherlands; 2 Bioprocess Technology Section (BPT), Department of Biotechnology, Faculty of Applied Sciences, Delft University of Technology, Julianalaan 67, 2628 BC Delft, The Netherlands; 3 Kluyver Centre for Genomics of Industrial Fermentation, Julianalaan 67, 2628 BC Delft, The Netherlands

## Abstract

In recent work, attempts have been made to link the structure of biochemical networks to their complex dynamics. It was shown that structurally stable network motifs are enriched in such networks. In this work, we investigate to what extent these findings apply to metabolic networks. To this end, we extend a previously proposed method by changing the null model for determining motif enrichment, by using interaction types directly obtained from structural interaction matrices, by generating a distribution of partial derivatives of reaction rates and by simulating enzymatic regulation on metabolic networks. Our findings suggest that the conclusions drawn in previous work cannot be extended to metabolic networks, that is, structurally stable network motifs are not enriched in metabolic networks.

## 1. Introduction

Metabolic networks are studied for a number of purposes, one of which is metabolic engineering, the optimization of industrial processes through directed genetic changes using recombinant DNA technology [1]. Another example is synthetic biology, "the engineering-driven building of increasingly complex biological entities for novel applications" [2]. These fields require the understanding of cellular function in detail, including the dynamics of all chemical compounds (metabolites) inside a cell. Kinetic models of metabolic networks provide a convenient and compact representation of the biochemical modifications (over time) of all chemical compounds in living cells (*metabolism*). These modifications are interesting because many phenotypic characteristics of a cell are determined by metabolites rather than by genes and proteins directly.

Unfortunately, the parameters of the kinetic models are very difficult to determine experimentally. Therefore, current analysis of metabolic networks relies mainly on structural information, available in the form of stoichiometry of the the chemical reactions. An example is provided by *Flux balance analysis* (see [3]). FBA allows us to determine the distribution of fluxes (i.e., reaction rates in steady state), assuming that the cell tries to optimize some objective (e.g., maximum biomass), and imposing constraints based on mass conservation and thermodynamics. This method, though extensively and successfully applied, does not provide any information about network dynamics (it links the stoichiometry to steady-state behavior). This is why in this paper, we try to infer dynamic properties of cell metabolism, based on the (local) structural information of metabolic networks, in terms of small network building blocks.

The biochemical interactions in large biological networks can be conveniently represented as directed graphs, in which the nodes represent the constituent building blocks (e.g., genes, proteins, metabolites, etc.), and the edges represent the interactions between them. These graphs can be decomposed into small subgraphs, called *network motifs*. The enumeration of all small network motifs (of three or four nodes) summarizes the local connectivity patterns of a large complex network. It has been shown that certain motifs are enriched (over-represented) in biological networks when compared to randomly constructed networks [4]. However, at present it is not clear what determines the particular frequencies of occurrence of network motifs in biological networks. One might hypothesize that some motifs possess properties important enough to entail evolutionary advantages, leading to relatively high occurrence. In [5], it has been investigated whether the stability of a motif is such a network property, by inspecting the correlation between over- or under-representation and a measure devised for *structural stability* of network motifs.

The method in [5] consists of two main steps: 

(i) calculate over- and under-representation of all motifs, that is, inspect which motifs occur more or less frequently in a biological network than would be expected by chance;

(ii) assign each motif a *structural stability score* (SSS); a motif is nothing more than a very small graph, containing no parameters that describe particular dynamics; the structural stability therefore assesses the fraction of parameter settings for which the motif is stable.

The data used in [5] consists of two transcriptional regulatory networks of *Escherichia coli* and *Saccharomyces cerevisiae*, a developmental transcriptional network of *Drosophila melanogaster*, the signal transduction knowledge environment (*STKE*) network, and a neural connection map of *Caenorabditis elegans*.

In recent work, the method described in [5] has met some criticism. In [6], it was argued that this work was too limited, since a single motif can exhibit a broad range of dynamic activity. Therefore, a motif cannot be simply classified by its structural stability. Furthermore, according to [7], structural stability is not an intrinsic property of biological networks; a network made up of a lot of structurally stable motifs is not necessarily stable itself. Therefore, it is not obvious why evolution should prefer structurally stable motifs. Moreover, when the baseline method of [5] is changed just slightly (by using a different null model for the generation of random instances of the given network), the enrichment of structurally stable motifs is lost.

However, according to [8], the design principles of *metabolic networks* differ from other biological networks. It was observed that motif enrichment profiles across metabolic networks are highly correlated, whereas this correlation between metabolic networks and other kinds of biological networks is much less. This motivated us to extend the analysis of [5] to metabolic networks, to test the hypothesis that structurally stable motifs are enriched in metabolic networks. This in turn could indicate that structural stability has driven the evolution of metabolic networks towards stable dynamic systems.

**Figure 1:**
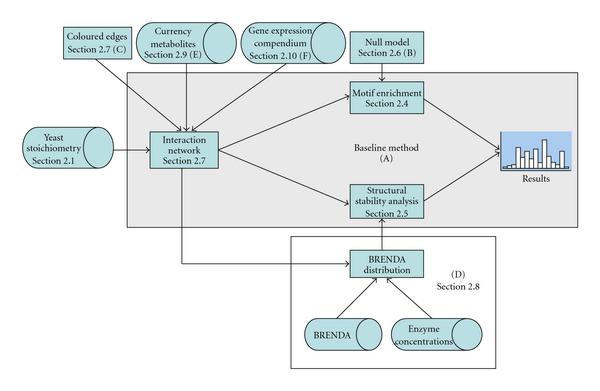
**Flowchart of the method proposed in this paper**. The blue area represents the baseline method as presented in [5], so method A from Table 1. The cylinder shapes represent data sources that are used, the rectangular shapes represents methods as described in the main text. The section numbers denote the method section in which the particular method is first described.

In order to make the proposed method more suitable for metabolic networks, we propose the extensions listed in the head row of Table 1. The flowchart in Figure 1 shows how our overall method results from the composition of the baseline method and the various additions.

**Table 1:** Paper overview

Method label	Null model	Colored edges	SSS	Currency metabolites	Enzymatic regulation	Result section	Method section
A	ER	No	Prill	Present	No	3.1	—
B	Switching	No	Prill	Present	No	3.2	2.6
C	Switching	Yes	Prill	Present	No	3.3	2.7
D	Switching	Yes	BRENDA	Present	No	3.4	2.8
E	Switching	Yes	BRENDA	Removed	No	3.5	2.9
F	Switching	Yes	BRENDA	Removed	Yes	3.6	2.10

The "method label" is used throughout the paper for reference. The "null model" indicates the type of network model (i.e., topology) used to generate random networks in the enrichment analysis (see Section 2.6). SSS is the structural stability score (see Section 2.5). "Currency metabolites" indicates whether these metabolites have been considered in the motif count (see Section 2.9). "Enzymatic regulation" indicates whether the actual enzymatic reactions activity has been taken into account (see Section 2.10). Finally, "result section" and "method section" list the sections in which the method is first described.

The baseline method calculated the enrichment score (called the *z-score*) of a motif by comparing the number of times it occurred in a real network as compared to randomized versions of this network. The collection of random networks is called the *null model*. The authors of [5] used the *Erdös-Rényi (ER)* method do randomize their networks. However, this method produces networks which have a Poisson degree distribution, whereas it is commonly observed that biological networks have degree distributions that follow a power law, that is, they are *scale-free* [9]. Therefore, we propose to use a different null model. The results change significantly.

The next addition deals with interaction types. Because of the nature of the networks used in [5], it was hard to determine whether an interaction between two nodes should be an activating (positive) or an inhibitory (negative) interaction. Determining this in metabolic networks is straightforward. We propose to subdivide each motif into a group of motifs of similar structure but different interaction types, represented by edge colors. This might provide more insight in particular motif enrichments.

**Figure 2:**
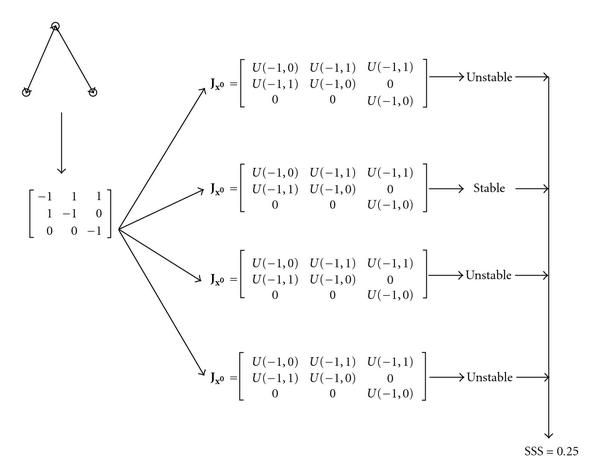
**Schematic overview of the process of calculating the SSS**. The motif in the top left corner is transformed to the adjacency matrix. Next, 10 000 Jacobians in steady state are generated by filling the nonzero entries of the adjacency matrix with values sampled from  and  distributions. In this figure, only four Jacobians are generated. Next the eigenvalues of all Jacobians are calculated. When all eigenvalues of a Jacobian are negative numbers with zero imaginary part, the steady state corresponding to that Jacobian is called (asymptotically) stable without oscillations. The SSS is the fraction of these steady states over all 10 000 steady states. Assuming that the four Jacobians in this example have the stability shown in the right of the figure, the SSS is 0.25.

In the second step in [5], the structural stability score (SSS) of a motif is assessed by a process which involves sampling from a uniform distribution (see Figure 2). The sampled values represent interaction strengths between nodes in the networks. For the datasets in [5], it was not possible to assess the magnitude of these interactions, which is why the interaction strengths were sampled from a  (uniform in the range −1 to 1) distribution. However, since this paper deals with a metabolic network, a distribution built on kinetic parameters associated to our specific network can be constructed, and we can change the uniform distribution to one that is biologically more plausible.

Metabolic networks contain a number of hubs (i.e., nodes with a very high degree compared to the great majority of nodes). These hubs are mostly cofactors, or *currency metabolites*, which typically are not the metabolites of interest in a reaction. These currency metabolites have a large number of interactions with other metabolites, whereas in reality these interactions are only indirectly present. Interactions with hubs in our modeled metabolic network lead to inflated motif frequencies for some motifs, which is why we remove currency metabolites from our network, using a similar approach as in [10].

The last addition to the method proposed in [5] deals with enzymatic regulation. Metabolic networks are often represented by their *stoichiometric matrix*, containing membership information of all metabolites in all reactions. However, in a cell, not all reactions are active all the time. The majority of reactions inside a cell are catalyzed by enzymes, which are in turn encoded for by genes. So the transcriptome can influence the metabolism of a cell by means of enzymatic regulation. We present a way to model this by using gene expression data.

## 2. Methods

### 2.1. Datasets

#### 2.1.1. Metabolic Network

We chose to study the *S. cerevisiae* metabolic model presented in [11]. This model not only contains a quite complete list of chemical reactions including compartmentalization information, but also the great majority of reactions are associated to genes. The authors provided us with a newer version of this model (*S.cerevisiae iMM904*), which remains to be published. See Table 2 for details.

**Table 2:** Summary of the data from [11]

Items	Number
Metabolites	1223
Irreversible reactions	929
Reversible reactions	477
Total number of reactions	1883

#### 2.1.2. Brenda

To construct the distribution to use in the structural stability analysis, we used the enzyme database BRENDA (http://www.brenda.uni-koeln.de). BRENDA is indexed with Enzyme Commission (EC) numbers, which is a classification scheme for enzymes. Each EC number specifies an enzyme-catalyzed reaction of which measurements of enzymatic parameters are listed.

#### 2.1.3. Microarray Data

We used the microarray compendium from [12], a large collection of microarray experiments performed under a number of different growth conditions. Each experiment lists three different values for a large number of genes, namely the microarray analysis suite (MAS) value [13], the robust multichip analysis (RMA) value [13], and a detection *P*-value [14]. These datasets are used to rank genes according to their expression profile over multiple conditions. This ranking in turn defines different instantiations of our metabolic network (see Section 2.10). Table 3 shows the dimensions of this dataset.

**Table 3:** Summary of the data from [12]

Items	Number
Experiments	165
Conditions	54
Genes	9335
Genes in [11]	902

### 2.2. Dynamic Systems and The Jacobian

A metabolic network is a dynamic system, which can be described by a set of differential equations [15]. For a metabolic network consisting of three metabolites the differential equations are(1)

where , and  are nonlinear functions of the vector of all metabolite concentrations . The behavior of this dynamic system around a steady state can be described by the *Jacobian* of the system:(2)

which is the square matrix of all first-order partial derivatives of a vector-valued function [15]. This allows the dynamic system to be written as: (3)

which is the first order Taylor expansion of the dynamic system in some state , a linear approximation of the system at state . Equation (3) describes the evolution over time of the vector of metabolite concentrations  (*trajectory*). Steady states are constant trajectories, hence they can be obtained as solution of (3), when the time derivative of the concentrations is set to zero (metabolite concentrations stay constant over time). For a more detailed explanation, see the Jacobian section in Supplementary Material available online at doi:10.1155/2009/630695.

### 2.3. Network Motifs

A metabolic dynamic system as described in Section 2.2 is represented by a metabolic network, which is a graph consisting of metabolites (nodes) and interactions (edges). A network motif is a very small directed subgraph. This work only deals with motifs consisting of three nodes of two to six directed edges (see Supplementary Figure 20 for the results with 4-node motifs). Since the pioneering work of Milo et al. [16], network motifs have been widely used to study the local topology of many different biochemical networks. In this paper, we have therefore chosen to use network motifs analysis also for metabolic networks (rather than devising alternative building blocks). Each motif in a metabolic network is a set of three connected metabolites that have interactions between them, such that the shortest path between any of the three metabolites is at most two. Because of this, one metabolite can be a member of multiple motifs. The same is true for an interaction.

Although sets of three metabolites might have very different interaction strengths, motifs are only concerned with structure, that is, the interaction between the metabolites is binarized, either there is an interaction or there is not. This leads to 13 possible network motifs (see Supplementary Figure 9(a)). The interaction information between all nodes (i.e., metabolites) of the motif is summed up in the *adjacency matrix*. Such a matrix has a nonzero entry at  when metabolite  is influenced by metabolite  (see Supplementary Figure 9(b) for an example). The adjacency matrix contains only the binarized interaction information between three metabolites, whereas the Jacobian contains the actual interaction strengths between the metabolites.

### 2.4. Motif Enrichment

Motif enrichment is determined by generating a large number of randomized versions of the original network, and calculating a *z-score* for each motif (4)

where  is the number of occurrences of motif  in the real network and  the set of occurrences of motif  in the random networks [17]. A high *z*-score for motif  indicates that the probability of finding motif  as often as in the real network by chance is low. Conversely, a large negative *z*-score indicates that the probability of finding motif  as little as in the real network is low. *Z*-scores are transformed into *normalized z-scores (NZS)* of unit length by using (5)

in which  is the number of motifs. These NZS can be compared across different networks.

### 2.5. Structural Stability

In Section 2.2, we have shown how to linearize a dynamic system (representing a metabolic network) around a steady-state . A steady state is *asymptotically stable*, when all the trajectories of the dynamic systems starting in a perturbed state (in a small neighborhood of the considered steady state) eventually converge to the steady state (while remaining bounded). A necessary and sufficient condition for asymptotic stability is that all the eigenvalues of the Jacobian have a negative real part. If the eigenvalues with a negative real part have a null imaginary part, the perturbed trajectories will converge to the steady-state without oscillations. In Prill et al. [5], the *SSS* is defined as a measure for the probability that the dynamical systems that can be associated to a given motif are locally (i.e., around a steady state) asymptotically stable with no oscillatory modes (such a condition is more restrictive than just demanding asymptotic stability). This score is determined by first generating a large number of possible Jacobian matrices for a given motif, and subsequently calculating the eigenvalues of each of these Jacobians matrices (see Figure 2). The SSS is the fraction of the Jacobians of which all eigenvalues have a negative real part and zero imaginary part. As it is computationally intractable to instantiate every possible Jacobian, we sample from the space of possible Jacobians, which is done by instantiating 100000 Jacobians in which each nonzero entry is sampled from a given distribution. In Prill et al. [5], Jacobians are constructed by assigning a value sampled from a  distribution (uniform over range (−1,1)) to all nondiagonal, nonzero entries and a value sampled from a  distribution to all diagonal entries of the adjacency matrix of a motif. Note that the range of the SSS is , with a value of 1 indicating that any dynamic system associated to the motif is stable (i.e., the interaction signs and strengths do not influence the stability). On the other hand, a low value indicates that only a small fraction of all possible parameters of the Jacobian can guarantee stability.

### 2.6. Method B: Random Networks

The calculation of the NZS (Section 2.4) requires randomizing networks and counting motifs. Both of these tasks are performed by the Mfinder and FANMOD programs [18, 19]. These two software tools yield the same results, but FANMOD can handle colored edges, whereas Mfinder cannot. In this work, two different methods of generating random networks are used: The *Erdös-Rényi (ER) method* and the *switching method* [20], which is the default method used by both Mfinder and FANMOD.

The ER method puts  nodes on a canvas and subsequently adds  directed edges, uniformly picked from the set of all possible  edges. Networks generated by this method have node degree distributions that follow a Poisson distribution, whereas it is commonly observed that biological networks are scale-free, that is, their node degree distribution follows a power law [21]. Random networks generated using this method are therefore deemed less suitable for representing biological networks. Another problem is that the probability of generating bidirectional edges is low; bidirectional edges are rare in ER networks. Network motifs with such edges are hardly found in ER random networks, resulting in low (zero) variance and thus in an infinite NZS, which is obviously an undesired effect.

The switching method on the other hand operates as follows switchingmethod. The original network is used as basis and a pair of edges () is repeatedly randomly selected and switched to obtain (). The exchange is only performed if it does not introduce an edge that already exists or a self edge, that is, an edge from a metabolite to itself. Furthermore, unidirectional edges are only exchanged with other unidirectional edges and bidirectional edges only with bidirectional ones. Edge "colors" (corresponding to some discrete property) can also be taken into account, that is, edges are only switched when they have the same color. The process is repeated a sufficient number of times for the random network to show good mixing (for details, see [18]). The switching method preserves the number of incoming, outgoing, and bidirectional edges of each node of the real network, and thus the exact degree distribution, making it a more reliable enrichment analysis in biological networks.

### 2.7. Method C: Network Structure Generation

It is not straightforward to model a metabolic network. Where it is quite clear what an edge in a transcriptional regulatory network means, namely the regulatory effect of one gene on another, it is less clear what the meaning is of such an edge between two metabolites in a metabolic network. We let edges represent influences between metabolites as they would occur in the Jacobian matrix of the dynamic system, (see (3)). In our case, the Jacobian has size  with  the number of metabolites. Each element  represents the influence of metabolite  on metabolite .

The following is built on the knowledge that for a metabolic network the Jacobian matrix  is given by(6)

in which  is a stoichiometric matrix of size , with  and  the number of metabolites and reactions, respectively, and  is a matrix of size  of partial derivatives of the vector of reaction rates  with respect to the vector of metabolite concentrations  in steady-state . A stoichiometric matrix contains the reaction coefficients of every chemical reaction in a network. Each reaction is represented by a column in the matrix in which substrates and products of that reaction have some negative or positive integer value, respectively. The metabolic network (Section 2.1.1) results in a stoichiometric matrix  with  and . Note that each of the 477 reversible reactions is represented as two unidirectional reactions.

**Figure 3:**
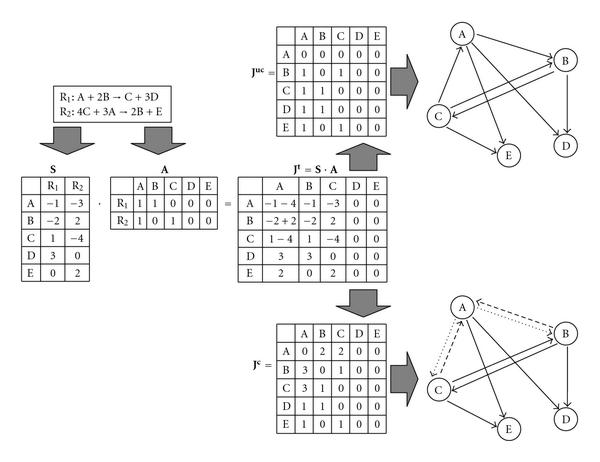
**The artificial network consisting of the two reactions shown at the top left of this figure is used to illustrate how matrices  and  are constructed**. Also, an intermediate matrix  (not described in the main text) is shown, which is the result of the multiplication . Matrix  is constructed by setting all diagonal entries to zero and putting ones at the locations where there are only positive values in . Matrix  is constructed in a similar manner for the diagonal and positive entries. However, the entries of  consisting of only negative values are set to 2, whereas the entries consisting of both positive and negative values are set to 3. A drawing of the resulting uncolored and colored networks is shown at the bottom right.

From the stoichiometric information in , a matrix  can be constructed which has the same dimensions as  and has an entry of 1 at position  when the partial derivative of reaction  w.r.t. the concentration of metabolite  is nonzero and 0 otherwise. When we substitute  in (6) by  we obtain a Jacobian which can be used to construct matrices  and  representing an uncolored and a colored model of our network, respectively (later, edge color will be used to differentiate different type of interactions).  is constructed such that  is 1 when metabolite  has a positive effect on metabolite , that is, metabolite  is a substrate in at least one reaction where metabolite  is a product, and 0 otherwise.  on the other hand, has three interaction types: positive, negative, and combined, which are (arbitrarily) represented by , and 3, respectively. A positive interaction is defined similar as for . A negative interaction at  indicates that metabolites  and  are both substrates in the same reaction. A combined interaction is a combination of these two interaction types, which should be thought of as an interaction which can act in both a positive and a negative way.  contains the structural information without interaction types, or colored edges. This network will from now on be referred to as the *uncolored network*. Conversely, the network represented by  will be referred to as the *colored network*. Figure 3 shows how a small artificial network consisting of only two reactions would be transformed into both networks.

### 2.8. Method D: B-Sss

In a metabolic network, entries in the Jacobian matrix represent interactions between metabolites. In our analysis, we assume that all reaction rates follow the Michaelis-Menten kinetic rate law:(7)

where  is the Michaelis-Menten constant,  is enzyme concentration and  represents the maximum number of moles of substrate that the enzyme can convert to product per unit time (see [22]).

Differentiating (7) gives (8)

We are interested in the values of  in order to calculate the matrix . Subsequently, these can be sampled from to generate matrix  (Section 2.7).

Values for  and  are collected by parsing BRENDA, whereas values for  are collected from Ghaemmaghami et al. [23], which contains a list of concentrations for a number of proteins of *S. cerevisiae*. Values for , and  were selected as triplets only when (i) the three values belong to the same protein; (ii)  and  correspond to the same substrate, and (iii)  and  correspond to the same conditions. Each triplet of values for  and  was entered 100 times in (8), each time using a value for  uniformly picked from the range  moles per liter Henry et al. [24]. The result is a distribution for , of which each sample represents an entry in the  matrix.

Next, 1000 matrices ,  are generated, by assigning all nonzero entries in  a value randomly sampled from the  distribution. Using these s, 1000 matrices  are generated(9)

Finally, all nonzero entries in the 1000 Jacobians thus generated are distributed over vectors , and , representing positive, negative, combined, or diagonal entries of a Jacobian matrix, respectively, such that , , , and . These four vectors together will from now on be referred to as the *BRENDA distribution*. This distribution consists of a large amount of small values with high variation and a few larger values (see the histograms of  and  in Supplementary Figures 14(c) and 14(d)).

In Section 2.5, it was discussed how the method described in Prill et al. [5] determines the SSS of a motif. It instantiates Jacobians by sampling values from uniform distributions and entering them in the adjacency matrix. We instead sample from the BRENDA distribution, which seems biologically more relevant. This is done by assigning diagonal values, nondiagonal values of 1, nondiagonal values of 2, and nondiagonal values of 3 in the adjacency matrix of a motif a value sampled from , , , or , respectively. The remaining part of the procedure is the same as described in Section 2.5, yielding a new structural stability score based on the BRENDA distribution. This new SSS and the SSS of Prill et al. [5] will from now on be distinguished as the *B-SSS* (for BRENDA) and the *P-SSS* (for Prill), respectively.

### 2.9. Method E: Currency Metabolites

Currency metabolites are chemicals that participate in a reaction but are not the chemicals of interest in that reaction. Their role is mostly to transfer energy, OH-groups or H-atoms. In [10], it was observed that these currency metabolites greatly influence the topology of a metabolic network (see Supplementary Figure 10). As it is likely that the way in which currency metabolites are treated also influences motif frequencies, they are removed from our network in order to assess their impact on motif enrichment.

However, care must be taken in deciding which metabolites to remove. Some authors have determined metabolites that frequently operate as cofactors [10, 25] by hand, whereas in [26] a method was developed where currency metabolites could be automatically identified based on the modularity of the network. Using the lists of currency metabolites from [10, 25, 26] as a starting point, currency metabolites are removed from our network in two steps. First we defined a set of metabolites which are always currency metabolites. These are removed from our network completely, that is, the rows of the stoichiometric matrix  corresponding to these metabolites are deleted (Supplementary Table 6).

There are also chemicals which are currency metabolites in one reaction but not in another. We observed that these metabolites usually come in pairs (Supplementary Table 7). We removed these pairs in all reactions in which one metabolite is a substrate, whereas the other is a product. The reactions that consisted *only* of these pairs of currency metabolites (Supplementary Table 8) were kept however, since these are the reactions in which the currency metabolites are created.

### 2.10. Method F: Enzymatic Regulation

#### 2.10.1. Preprocessing

Recall from Section 2.1.3 that our microarray data consists of the expression of 9335 genes over 165 experiments using three different ways of normalization (Table 3). We selected only genes known to regulate reactions in our network, leaving us with 902 measurements per experiment. It appeared that some experiments structurally showed higher expression values for all genes. We exclude these experiments from our data because in subsequent sections, we normalize gene expression by dividing by their maximal expression over all experiments. The average number of genes having their maximum expression level in a particular experiment is 5.4, with a standard deviation of 8.9 (see the histogram in Supplementary Figure 14(f)).

All experiments that had a value higher than  were deleted from the compendium, leaving us with 154 experiments. After deletion of these experiments, 53 different conditions remained. In order to avoid a bias toward setups that occurred more frequently than others, we averaged the expressions of all experiments belonging to the same setup, resulting in 53 expression values per gene. As described in Section 2.1.3, our microarray data consists of three types of values: absolute expression using RMA and MAS normalization, and detection *P*-values.

#### 2.10.2. Mas and Rma

We incorporate the method as described in [27], in which we assume that every gene is expressed in at least one condition. We look for the maximum value of a gene over all conditions and consider that a value for which the gene is expressed. Subsequently, we divide all other expressions of the same gene by this maximum value, thus normalizing to a range between 0 and 1. In conditions where this normalized value is close to zero, we assume that the gene is not expressed. This can be done for all genes and by defining a threshold , lists can be created for all conditions, containing all genes that have a normalized expression below .

#### 2.10.3. Detection of *P*-Value

The smaller the *P*-value, the more likely it is that the particular gene is expressed in a particular condition. We define a threshold, , but here we create lists for all conditions containing genes with a *P*-value above this threshold.

#### 2.10.4. Removal of Reactions

The generated lists contain the gene names corresponding to reactions in our metabolic network. The columns of stoichiometric matrix  corresponding to these reactions are removed and thus the metabolite interaction that would have resulted from these reactions will not appear in matrix , which represents the colored network. After this processing, we end up with 53 different stoichiometric matrices for each method-threshold combination. Now the analysis continues as usual; for each stoichiometric matrix, a motif enrichment analysis is performed and the NZS-profiles over all conditions are averaged for every method-threshold combination, providing us with results that can be directly compared to those obtained from the full model in which no reactions were deleted.

## 3. Results and Discussion

### 3.1. Results of The Baseline Method Differ across Networks

**Figure 4:**
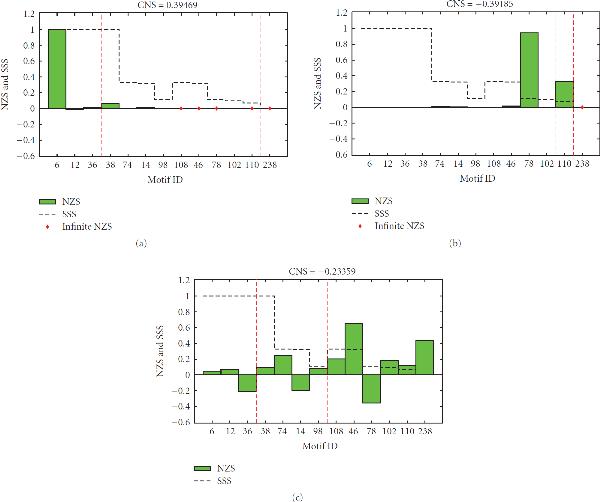
**Relation between stability (measured by the *SSS*) and motif enrichment (measured by *Normalized Z-Score (NZS)*)**. Motifs are first sorted according to density classes (separated by red dashed lines), groups of motifs having an equal number of edges. Inside each density class, motifs are sorted by descending SSS. The first class consists of motifs with 2 edges, the last class of the fully connected motif with 6 edges (see Supplementary Figure 9(a) for the 13 motifs). Motifs that are infinitely enriched, resulting from a division by zero in (4), are represented by a red diamond at . Where neither a green bar nor a red diamond can be seen, the NZS is very close to zero. The title of each figure shows the *Correlation between NZS and SSS (CNS)*, a quantitative measure for the enrichment of structurally stable motifs. (a) Example of a result from [5], organism: *S. cerevisiae*. (b) Method A from Table 1 on the metabolic network of [11]. (c) Method B from Table 1 on the metabolic network of [11].

Figure 4(a) shows a representative result presented in [5] (an overview of all their results is given in Supplementary Figure 14(a)). The *x*-axis shows the motifs, and the *y*-axis shows both the SSS and the NZS. The motifs are divided into *density classes*, groups of motifs having an equal number of edges. In the case of 3-node motifs, the number of edges ranges from 2 to 6, yielding 5 density classes. For each figure, the *Correlation between NZS and SSS (CNS)* is given, which is a quantitative measure for the enrichment of structurally stable motifs. When the CNS is high (close to 1), stable motifs are enriched, when it is low (close to −1) unstable motifs are enriched. In [5], a descending stairs-like behavior was observed, that is, within each density class, the highest scoring motifs appear on the left, the lower scoring motifs on the right. This means that within each density class there is a positive correlation between the SSS and the NZS of a network motif, which led the authors of [5] to the conclusion that evolution has selected for stable motifs.

When the same method (method A from Table 1) is applied on our metabolic network, we obtain the results in Figure 4(b), which do not correspond to those in [5]; structurally stable motifs are not enriched. Only two motifs are highly enriched, 78 and 110, which both have an infinite NZS in Figure 4(a). This results from a division by zero in the calculation of the NZS, as it is discussed in detail in Section 2. The motif most enriched in Figure 4(a), motif 6, is not over-represented at all in our network. Motif 78 consists of two bidirectional edges (Supplementary Figure 9(a)). The large difference in enrichment scores for this motif stems from the fact that the Erdös-Rényi (ER) randomisation method used here produces very few bidirectional edges (Section 2.6). Motif 78 was generated only a few times in the random networks for our metabolic network, resulting in a low standard deviation in the equation calculating the NZS (Equation (4) in Section 2.4), and thus a high enrichment. In the random networks of the example taken from [5], the motif was not generated at all, resulting in an infinite enrichment, indicated by the red diamond. Motif 238, consisting of even more bidirectional edges, is never generated and is thus infinitely enriched in both networks. Finally, note that motif 78 is not the most stable motifs in its density class.

To summarize, Figures 4(a) and 4(b) show that when applying the baseline method of [5] on a transcriptional regulatory network and a metabolic network (both of *S. cerevisiae*), very different results are obtained. It is likely that this difference is caused by the method used for generating random networks. Therefore, the influence of the null model on our analysis is inspected in Section 3.2. Furthermore, there is no stairs-like behavior in our results, leaving us with little evidence for the central hypothesis given at the beginning of this paper.

### 3.2. The Choice of Null Model Greatly Influences Motif Enrichment Results

If we change the null model from the ER-method to the switching method (Section 2.6), we obtain the results shown in Figure 4(c). It is clear that the choice of randomization method has considerable influence on the results of our metabolic network. The top scoring motif in Figure 4(b), motif 78, now has the lowest NZS, caused by a high frequency in the random networks. Motif 238 has a quite high NZS, but no longer infinite as in Figure 4(b). These observations indicate that the switching method has generated more bidirectional edges than the ER method, as expected.

The top scoring motif in the new results is now motif 46. This is the motif with the highest SSS of its density class. However, as this is the only motif for which this is the case, we cannot conclude that the structural stability of network motifs has driven the evolution of our metabolic network. We have also performed method B (Table 1) on some of the data of [5], which led to similar results (Supplementary Figure 13).

In summary, by replacing the ER randomization method by the switching method, we obtained results for the metabolic network which are almost completely opposite to the original ones, that is, over-represented motifs are now under-represented and vice versa. The hypothesis that structurally stable motifs are enriched cannot be confirmed yet.

### 3.3. Colored Edges Give A More Detailed Picture of Motif Enrichment

**Figure 5:**
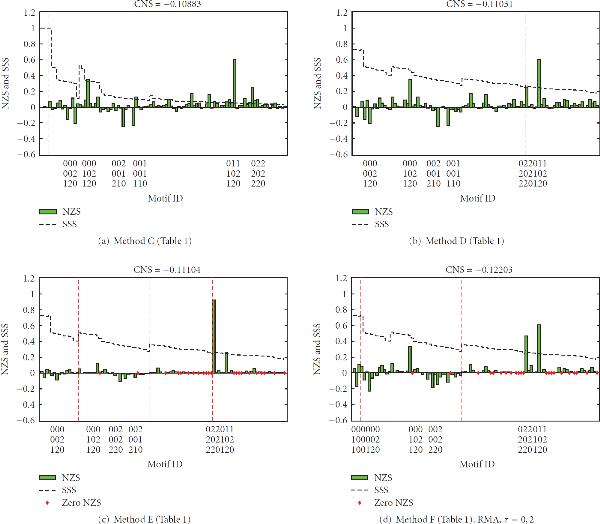
**Results of using method C (a), D (b), E (c) and F (d) from Table 1**. Note that in (c) and (d) a number of motifs have disappeared from the real network, resulting in a zero NZS, depicted by the red diamonds.

The previous results were based on the 13 possible 3-node motifs of the uncolored model  (Section 2.7), in which interaction types were not taken into account. From this section on, we use the colored model , extending the uncolored model by assigning each edge a label of , or 3, indicating a positive, negative, or combined interaction (Section 2.7). When we bring these interaction types into use, the NZS profiles change in the sense that we now have NZS for 97 motifs instead of only 13. As the results in [5] only contain NZS profiles for 13 motifs, the results can no longer be compared directly. However, a similar plot (NZS versus SSS of motifs divided over density classes) can still be created (see Figure 5(a)). A number of density classes show some correlation between NZS and SSS. For instance, inside classes 3 and 5 the over-represented motifs generally appear on the left, whereas the under-represented ones appear on the right. However, the other classes do not show this trend, leaving us with little proof for the central hypothesis.

### 3.4. Using Biologically Plausible Interaction Strengths Leads to Less Variation in Results

The observation that sampling interaction strengths from a uniform distribution does not correspond well to modeling biological interaction strengths calls for a more natural way to sample edges in our network. In Section 2.8 we show how we build a distribution which is biologically more relevant for our metabolic network. Using values derived from the BRENDA database, a new structural stability score, called the *B-SSS*, is constructed (see the histograms of the *BRENDA distribution* in Supplementary Figures 14(c) and 14(d)).

**Figure 6:**
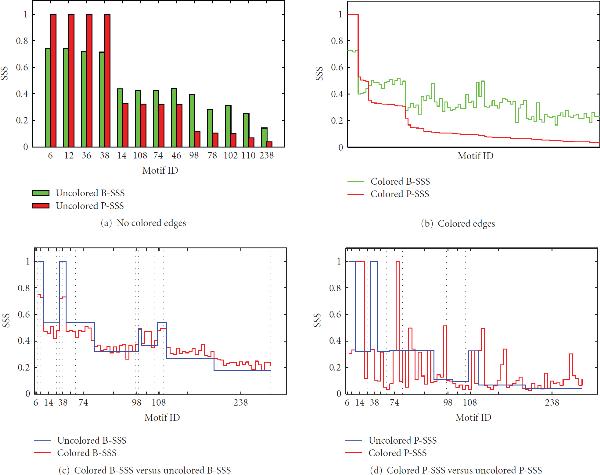
**(a) and (b) represent comparisons of the B-SSS and the P-SSS**. In both plots, the SSSes are ranked according to descending P-SSS. Note that the original P-SSS (for 13 motifs) cannot be compared directly to our B-SSS (for 97 motifs). We defined four SSSes: colored P-SSS, uncolored P-SSS, colored B-SSS, and uncolored B-SSS. The uncolored P-SSS is the original SSS from [5], the colored B-SSS is the SSS discussed in Section 2.8. The colored P-SSS is calculated by sampling positive, negative, and combined interactions from a , and a  distribution, respectively. The uncolored B-SSS is calculated by sampling each nonzero, nondiagonal entry and each diagonal entry in the adjacency matrix of a motif from the  and  distribution given in Section 2.8, respectively. Note that in (a) the B-SSS is always higher than the P-SSS except for the first four motifs, which have a P-SSS of 1, that is, they are always stable, no matter the interaction strengths. (b) shows the same behavior. The difference between the red and green lines is a measure for the change in structural stability for each motif. Therefore, the fluctuation in the B-SSS tells us that some motifs are more affected by changing the distribution used to sample from than others. (c) and (d) represent the effect of edge colors on the two SSSes. The 75 different motifs are sorted according to their ID. The black dashed lines divide the *x*-axes into 13-motif segments. A segment contains all motifs which have the same ID, and thus structure, but different edge colors, that is, interaction signs. The red lines are the SSS scores for the 97 different motifs, the blue line represents the original SSS score for each motif id. (c) The red line is the B-SSS of the colored model (, Section 2.7), the blue line is the B-SSS of the uncolored model (). (d) The red line is the P-SSS we have created for the colored model, the blue line is the original SSS from [5]. The red line in (a) follows the blue line quite closely, whereas in (b) the similarity is smaller.

Figures 6(a) and 6(b) compare the SSS as proposed in [5], *P-SSS*, and the B-SSS. It can be seen that there is quite some difference between the two scores. Another observation is that the B-SSS never becomes 1, that is, according to this SSS there are no motifs that are always stable, no matter the interaction strength. This is in contrast to the P-SSS, where motifs , and 38 are always stable.

Figures 6(c) and 6(d) compare the SSS profiles for the colored and the uncolored model as described in Section 2.7. It can be observed that the difference between the B-SSSes for the colored and the uncolored model is smaller than for the P-SSS. The colored B-SSS stays close to the uncolored B-SSS, whereas the colored P-SSS oscillates around the uncolored P-SSS. Note that the black dashed lines define regions consisting of motifs having adjacency matrices with the same nonzero entries. The signs of these entries differ however. From these results we can conclude that when the B-SSS is used, the sign of an edge is less important in determining stability than when the P-SSS is used. It could be that the uniform distribution used in the P-SSS generates Jacobians that are not biologically plausible, whereas the BRENDA distribution, which consists of interaction values derived from a database of measured values, does not. So motifs are less sensitive to changes in the signs of their interactions than the P-SSS suggests.

The main observation is that the P-SSS has a wider range than the B-SSS. It can be argued that this range is an artefact of the uniform distribution from which interactions are sampled and that the B-SSS limits the stability of the motifs to a smaller range.

The relatively small influence of the change of SSS on the enrichment analysis can be seen when we compare Figures 5(a) and 5(b). The overall conclusions remain the same, although zooming in on the last density class at the right of Figure 5(b) shows that the NZS do show an increased stairs-like behavior; the more stable motifs within the last density class are over-represented.

### 3.5. Removing Currency Metabolites Significantly Changes The Enrichment of Some Motifs

Currency metabolites are removed by the method described in Section 2.9. Removing currency metabolites has a large impact on the network topology. In particular, the degree distribution in the reduced network is not scale-free as in the complete network (see Supplementary Figures 11(a) and 11(b)). Note that although our reduced network is no longer scale-free, this has no consequences for the use of the switching method for random network generation. This method preserves the precise degree distribution for any network and is thus not biased towards a scale-free or any distribution.

**Figure 7:**
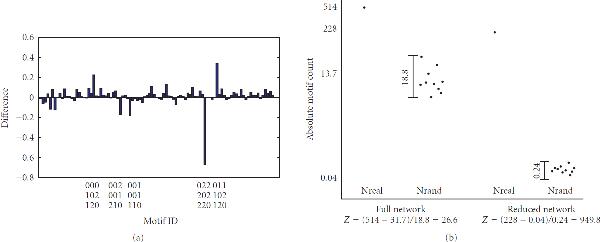
**(a) NZS of the full model minus NZS of the reduced model**. Observe the large negative peak on the right of the plot, which belongs to the motif with the adjacency matrix in (10). (b) Schematic drawing of the explanation for the large negative peak in (a). The *y*-axis is not to scale. The values on the *y*-axis and the *z*-scores on the *x*-axis are the actual values as calculated by FANMOD.

**Figure 8:**
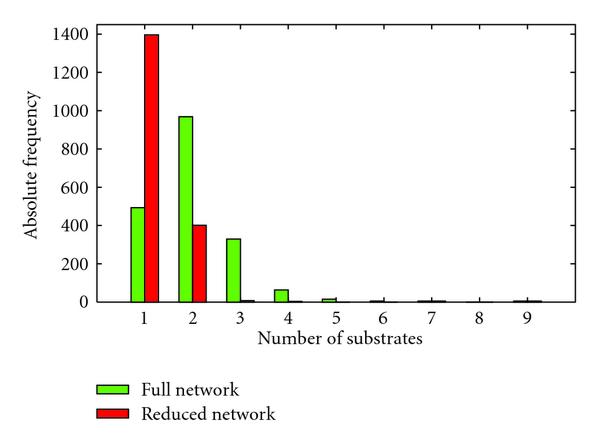
**Number of reactions with a certain number of substrates for both the full and the reduced network**. Removing currency metabolites causes the number of reactions having more than 2 substrates to drop significantly.

Figure 7(a) shows the difference between the two sets of NZS resulting from motif enrichment analysis on both the full and the reduced network (see Supplementary Figure 14(e) for the separate NZS). The ordering of the motifs is the same as in Figure 5(b). There is quite some difference between the two scores. The most striking result is the high negative peak on the right of Figure 7(a), corresponding to the motif with adjacency matrix(10)

which is the fully connected motif consisting of only negative interactions. The peak tells us that the probability of finding this motif by chance as often as in the real reduced model is far lower than the probability of finding it in the real full model. A schematic drawing of this observation is shown in Figure 7(b). Apparently, when currency metabolites are removed, the number of times this motif is generated in randomized versions of the reduced network drops faster than the frequency of this motif in the real reduced network. This might stem from the fact that the probability of generating this motif in random networks is dependent only on the number of negative bidirectional edges in our original network, whereas the frequency of the motif in the original network is guided by the format of the reactions. Any reaction with more than two substrates directly constructs such motifs, see Table 4. Removing currency metabolites decreases the overall number of substrates, thus significantly reducing the number of reactions having more than two substrates (Figure 8). As a result, the motif in (10) is not constructed as much as in the full network. This causes the frequency of this motif in the reduced network to drop significantly, whereas the random networks are not affected by this "direct motif construction."

**Table 4:** This table shows for each of the numbers of substrates involved in a single reaction (top row) how many fully connected motifs consisting of only negative interactions as in (10) are created in the network

Number of substrates	1	2	3	4	5	6	7	8	9
Number of motifs	0	0	1	4	10	20	35	56	84

One can see that removing substrates greatly decreases the number of this motif in the real network.

Figure 5(c) shows the effect of removing currency metabolites on our total NZS analysis. This figure should be compared to Figure 5(b). All NZSes have shrunken towards zero, both the positive and the negative ones. In the last density class, the stairs-like behavior is somewhat lost again. Although the highest peak is the most stable motif of its density class, removing currency metabolites, does not allow us to validate the central hypothesis.

### 3.6. Enzymatic Regulation Can Change A Metabolic Network's Building Blocks

We simulated enzymatic regulation using the three methods described in Section 2.10. Figure 5(d) shows the result of the motif enrichment analysis of the network where reactions have been deleted according to the RMA method with a threshold . For the complete set of 18 plots resulting from 6 thresholds for each 3 methods, see Supplementary Figures 15–19 (these should be compared to Figure 5(c)).

One can immediately see that the introduction of enzymatic regulation does not change the overall NZS-profiles dramatically. For low  and for high , a lot of motifs that were present in the real network of Figure 5(b) disappear from the NZS-profile (see Supplementary Figures 15, 16, and 17). The network decreases significantly in size (using the RMA method with , 1017 reactions are discarded), so there are less motifs in total. This causes very infrequent motifs in the unregulated network to disappear first.

The peaks in the plots always remain at the same positions. In the RMA plots, the two largest peaks switch for some thresholds, but this is not a general trend; when the threshold becomes very large, the peaks are found again at the same places as in the unregulated network. The largest peak of Figure 5(c) lowers quite dramatically even when only a few reactions are discarded. This is caused by the fact that reactions with a lot of substrates are switched off earlier than reactions with only a few substrates (see Supplementary Figure 12). This could stem from the fact that reactions having a large number of substrates also need a large number of enzymes to catalyze them. We have assumed that the removal of any single one of these enzymes results in the reaction being switched off. This could explain the fast decrease in the large peak as it corresponds to fully connected motifs with only negative edges which, as was discussed in Section 3.5, are generated directly by reactions having more than two substrates.

In conclusion, the overall NZS profiles remain unaltered when enzymatic regulation is simulated. However, these profiles have been averaged over all experimental conditions; there may be some variation between different conditions (Supplementary Figure 19 shows the motif-enrichment analysis for some method-threshold combinations of the two conditions that had the lowest correlation in NZS profiles).

## 4. Conclusion

We have attempted to bridge the gap between the available topological information on a metabolic network and its complex dynamical behavior, by using the method proposed in [5]. The hypothesis is that structurally stable motifs have driven network evolution, and that a large network consisting of small stable building blocks will show stable behavior itself. We have altered this method to make it better suitable for analysis of metabolic networks, by changing the null model for determining motif enrichment, that is, the way of generating random networks; using interaction types; putting a different distribution to determine the structural stability of a motif into use; removing currency metabolites; and finally by simulating enzymatic regulation.

The first conclusion was that the choice of null model in the method of [5] significantly influences the results of the analysis. Based on the high number of infinite NZSes obtained by using the Erdös-Rényi method, we conclude that this method produces doubtful motif enrichment results due to its too random distribution of edges in random networks.

We have shown that the SSS proposed in [5], based on sampling interaction strengths from a uniform distribution, can give a false indication of the stability of network motifs in any network. We composed a biologically more plausible distribution for metabolic networks, the BRENDA distribution, and demonstrated that the SSSes constructed from this distribution have a smaller range than those constructed from a uniform one. Furthermore, using the BRENDA distribution, there is less variation in structural stability between motifs that have the same structure but different interaction signs.

In addition, we have performed an extensive simulation of enzymatic regulation, by removing reactions based on gene expression data obtained by three different methods. We have demonstrated that even averaged over a broad range of different experimental setups influencing gene expression, the motif enrichment profiles stay remarkably similar. This could indicate that the "building blocks" available to a cell to build its metabolic network from stay largely unaltered.

The main conclusion of this paper is that structurally stable network motifs are not enriched in metabolic networks. Even after applying a number of adaptations to make the method more suitable for metabolic networks, we were not able to use the method proposed in [5] to show a positive correlation between motif enrichment and stability in metabolic networks. The measure we have devised to quantify this correlation, the CNS, did not increase after the various adaptations, indicating that stable motifs are not enriched. In order to strengthen this conclusion, we repeated our analysis using larger motifs (4 nodes) and metabolic networks from other organisms (*E. coli* and *H. sapiens*) with identical conclusions. The results can be found in Supplementary Figure 20.

The extensions proposed in this paper are not exhaustive. However, we do not believe that further extensions would change the conclusions we reached in this paper. In fact, the results of the different extensions are consistent (i.e., structurally stable motifs are not enriched in metabolic networks), in spite of the fact that the topology of the metabolic network can be drastically changed in different ways.

The analysis proposed in this paper is statistical in nature, and so is the method proposed in [5]. We are not able to quantify the influence of the uncertainties of the considered models on the presented results. However, we believe that this work improves the method in [5], since we have considered biologically meaningful parameter ranges, whereas in [5], the parameters are drawn from uniform distributions; and we have used the information available about the interaction type and activity, whereas in [5], only binary interaction information is taken into account.

We can conclude that, in terms of deriving stability from structural properties, metabolic networks differ from the types of biological networks studied in [5]. It may be the case that metabolic networks indeed are less stable. However, we have focused on local stability only, and as measuring global stability is hard, this conclusion is not easy to validate. Perhaps a different global measure, such as monotonicity [28, 29], may give more insight into differences between metabolic and other networks. A second and more likely explanation is that metabolic networks differ in their topology from other networks to the extent that the method of analysis used based on motifs fails. This conclusion is supported by the fact that the method in [5] was shown to be sensitive to the choice of randomization; sensitivity to the structure of the input network is likely.

## Supplementary Material

## Supplementary Material

Supplementary Material The supplementary material encompasses (i) the list of symbols and notation used throughout the text, (ii) three tables clarifying the selection of currency metabolites, (iii) a list of figures illustrating to a full extent all the results we have obtained, and, finally, (iv) some explanations about the units used in the equations. More in detail, Figures 9–12 provide extra information about network motifs, currency metabolites, and topological features of the metabolic networks (e.g., degree distribution), whereas Figures 13–20 show the same kind of plots that can be found in the main text, namely, graphs representing the structural stability scores superposed to the normalized z-scores measuring motif abundance (the supplementary figures are relative to different methods for the evaluation of gene activity over different conditions). Click here for file
