# Supplementary material for: Stability from Structure: Metabolic Networks Are Unlike Other Biological Networks
Source: EURASIP J Bioinform Syst Biol. 2008 Dec 17;2009(1):630695. doi: 10.1155/2009/630695 (PMC3171437; doi:10.1155/2009/630695)
Supplement: Supplementary Material — The supplementary material encompasses (i) the list of symbols and notation used throughout the text, (ii) three tables clarifying the selection of currency metabolites, (iii) a list of figures illustrating to a full extent all the results we have obtained, and, finally, (iv) some explanations about the units used in the equations. More in detail, Figures 9–12 provide extra information about network motifs, currency metabolites, and topological features of the metabolic networks (e.g., degree distribution), whereas Figures 13–20 show the same kind of plots that can be found in the main text, namely, graphs representing the structural stability scores superposed to the normalized z-scores measuring motif abundance (the supplementary figures are relative to different methods for the evaluation of gene activity over different conditions). [file 1687-4153-2009-630695-S1.pdf]

5 Supplementary material

5.1 List of symbols

Symbol	Description
$\mathbf{x^0}$	Steady state
$\mathbf{J}$	Jacobian matrix
$\mathbf{S}$	Stoichiometric matrix
m	Number of metabolites
r	Number of reactions
$\frac{\partial v}{\partial x}\big\rvert_{x^0}$	Partial derivatives of v over x in steady state x_0
$\mathbf{A}$	Structural version of $\frac{\partial v}{\partial x}\big\rvert_{x^0}$
$\mathbf{J^{uc}}$	Jacobian matrix containing the uncolored network
$\mathbf{J^c}$	Jacobian matrix containing the colored network
v	Reaction rate
x	Substrate concentration
K_m	Michaelis-Menten constant
v_{max}	Maximum reaction rate
k_{cat}	Maximum number of moles of substrate that
	the enzyme can convert to product per unit time
$[E]$	Enzyme concentration
$\boldsymbol{j}_{pos}$	Vector containing the positive part of the BRENDA distribution
$\boldsymbol{j}_{neg}$	Vector containing the negative part of the BRENDA distribution
$\boldsymbol{j}_{com}$	Vector containing the combined part of the BRENDA distribution
$\boldsymbol{j}_{diag}$	Vector containing the diagonal part of the BRENDA distribution
i, j	Indices in any matrix
l	Index in the p Jacobians

Table 5: List of symbols

6 Jacobian

Consider the following hypothetical dynamic system given by the differential equations:

$$\begin{aligned} \frac{dx_1}{dt} &= f_1(\mathbf{x}) = x_1 \\ \frac{dx_2}{dt} &= f_2(\mathbf{x}) = 7x_3^2 \\ \frac{dx_3}{dt} &= f_3(\mathbf{x}) = 5x_2^3 - 4x_1 \end{aligned} \tag{11}$$

The Jacobian matrix $\mathbf{J}$ will be

$$\mathbf{J} = \begin{bmatrix} \frac{\partial f_1(\mathbf{x})}{\partial x_1} & \frac{\partial f_1(\mathbf{x})}{\partial x_2} & \frac{\partial f_1(\mathbf{x})}{\partial x_3} \\ \frac{\partial f_2(\mathbf{x})}{\partial x_1} & \frac{\partial f_2(\mathbf{x})}{\partial x_2} & \frac{\partial f_2(\mathbf{x})}{\partial x_3} \\ \frac{\partial f_3(\mathbf{x})}{\partial x_1} & \frac{\partial f_3(\mathbf{x})}{\partial x_2} & \frac{\partial f_3(\mathbf{x})}{\partial x_3} \end{bmatrix}, \tag{12}$$

which in this case would yield

$$\mathbf{J} = \begin{bmatrix} 1 & 0 & 0 \\ 0 & 0 & 14x_3 \\ -4 & 15x_2^2 & 0 \end{bmatrix} \tag{13}$$

When we substitute x_2 and x_3 for metabolite concentrations belonging to some point, $\mathbf{J}$ represents the best linear approximation of the dynamic system near that point.

7 Supplementary tables

Metabolite
H
H_2O
Phosphate
Diphosphate
CO_2

Table 6: Currency metabolites that were removed completely.

Metabolite 1	Metabolite 2
ATP	ADP
ADP	AMP
ATP	AMP
UTP	UDP
UDP	UMP
UTP	UMP
GTP	GDP
GDP	GMP
GTP	GMP
CTP	CDP
CDP	CMP
CTP	CMP
TTP	TDP
TDP	TMP
TTP	TMP
NAD	NADH
NADP	NADPH
Acetyl-CoA	Coenzyme A
Propanoyl-CoA	Coenzyme A
L-Glutamine	L-Glutamate
L-Glutamate	2-Oxoglutarate

Table 7: Currency metabolites that were removed in pairs

Reaction	Subsystem
(2) accoa → aacoa + coa	Fatty Acid Biosynthesis
(2) accoa → aacoa + coa	Fatty Acid Biosynthesis
akg + gln-L + h + nadh → (2) glu-L + nad	Glutamate metabolism
amp + atp ↔ (2) adp	Nucleotide Salvage Pathway
amp + atp ↔ (2) adp	Nucleotide Salvage Pathway
amp + gtp ↔ adp + gdp	Nucleotide Salvage Pathway
amp + gtp ↔ adp + gdp	Nucleotide Salvage Pathway
atp + cmp ↔ adp + cdp	Nucleotide Salvage Pathway
atp + gdp ↔ adp + gtp	Nucleotide Salvage Pathway
atp + udp ↔ adp + utp	Nucleotide Salvage Pathway
atp + cdp ↔ adp + ctp	Nucleotide Salvage Pathway
atp + ump ↔ adp + udp	Nucleotide Salvage Pathway
atp + ump ↔ adp + udp	Nucleotide Salvage Pathway
atp + gmp ↔ adp + gdp	Purine and Pyrimidine Biosynthesis

Table 8: These reactions consist only of the currency metabolites in Table 7. These are the reactions where currency metabolites are synthesised. Therefore, these reactions are not deleted from our network.

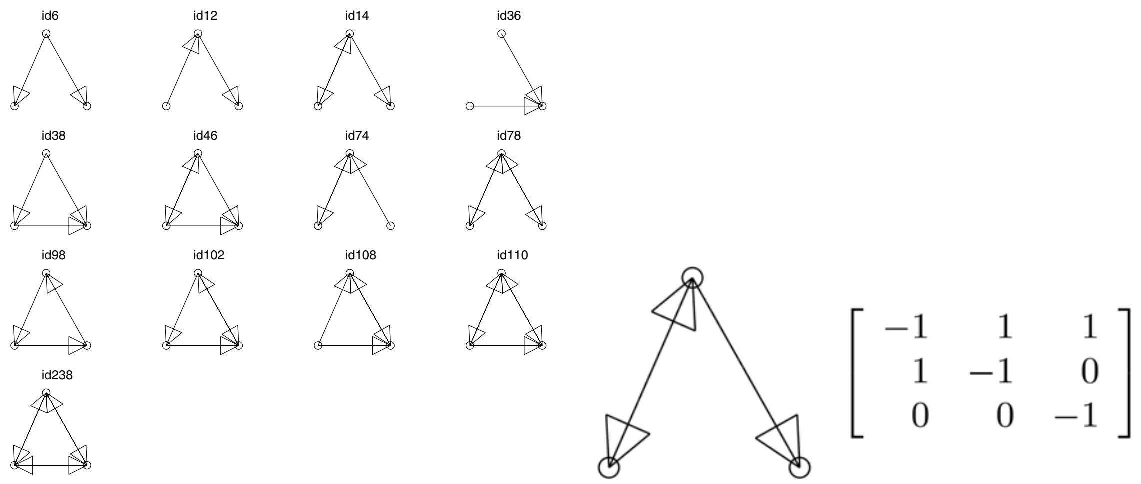

(a) (b)

Figure 9: (a) All possible network motifs consisting of 3 nodes. The motif ID is the decimal form of the binary integer that is be obtained by concatenating the rows of the adjacency matrix [Kashtan et al., 2004]. (b) Example of a 3-node motif and its adjacency matrix. Note the −1 values on the diagonal, which represent the self-degrading coefficients commonly observed in nature [Prill et al., 2005].

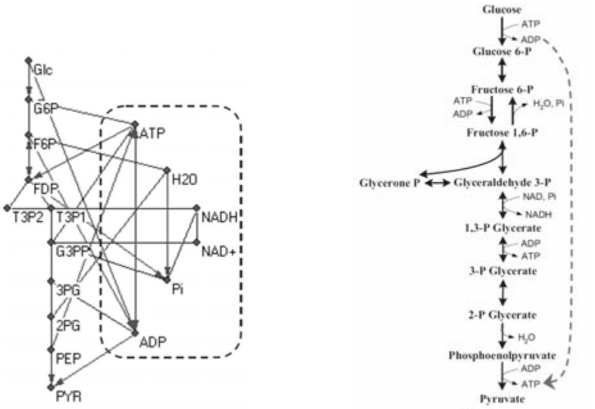

(a) Currency metabolites treated as nodes

(b) Currency metabolites not treated as nodes

Figure 10: The glycolysis pathway ([Ma and Zeng, 2003]). (a) Conventional way of representing a metabolic pathway. (b) The same pathway but with currency metabolites treated as nodes. It can be observed that the shortest path length from glucose to pyruvate is nine in (a) and two in (b).

8 Supplementary figures

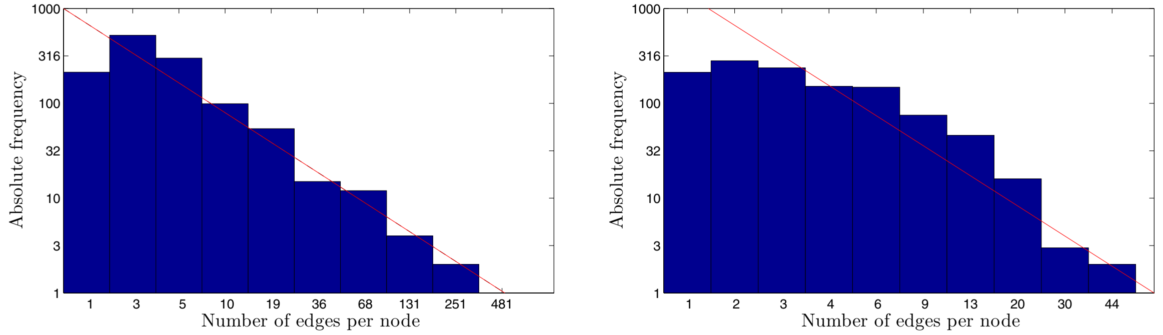

(a) In- and out-degree with currency metabolites. Scale: $\log_{10}$ vs. $\log_{10}$.

(b) In- and out-degree without currency metabolites. Scale: $\log_{10}$ vs. $\log_{10}$.

Figure 11: Histograms of node degrees. The histogram in (a) follows the straight red line (power-law distribution) closer than the one in (b), indicating that the full network is closer to being scale-free than the reduced one. Moreover, the most connected metabolite in (a) has a degree of 666, whereas in (b), the highest degree is 54.

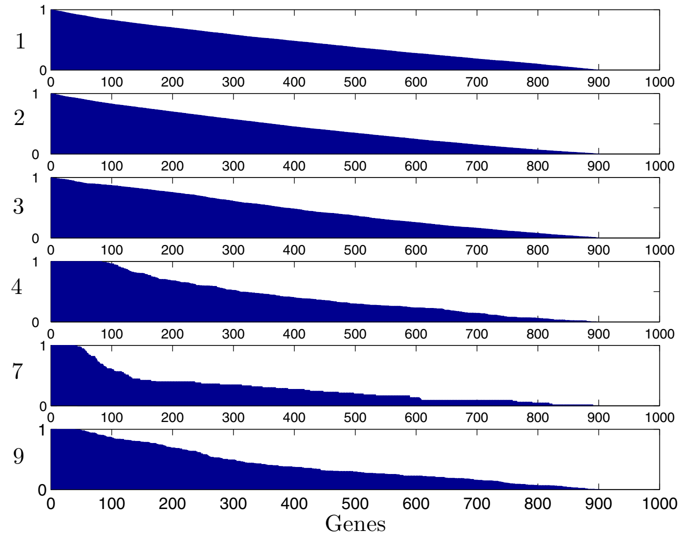

Figure 12: Removal of reactions with different numbers of substrates. The gene with lowest RMA-expression was removed iteratively. Then for each reaction type (i.e. reactions having the number of substrates indicated at the left of the plot), the fraction of reactions left over in the regulated network compared to the total number of reactions in the full network was plotted. The number of reactions with one, two and three substrates decreases quite gradually to zero, whereas reactions having four and especially five substrates show a steep descent when the first 200 enzymes are discarded. Note that a reaction with five substrates creates ten fully connected motifs consisting of only negative edges.

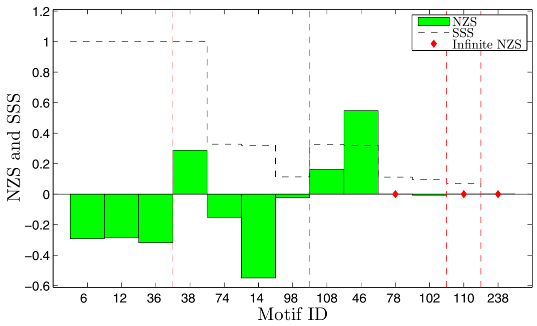

Figure 13: Method B from Table 1 on a the data of [Prill et al., 2005]. Organism: *S. Cerevisiae*.

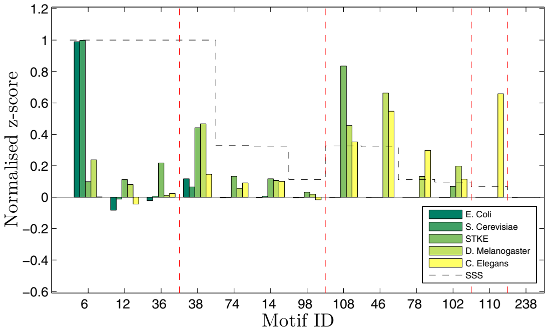

(a) Summary of the results in [Prill et al., 2005].

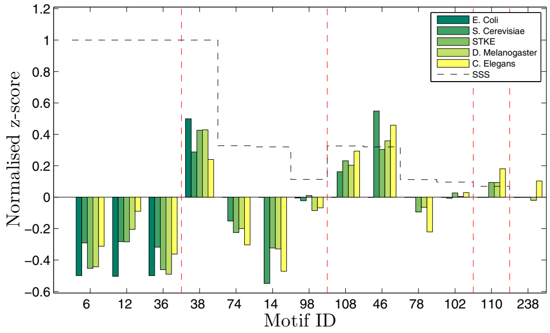

(b) Mfinder on Prill's data

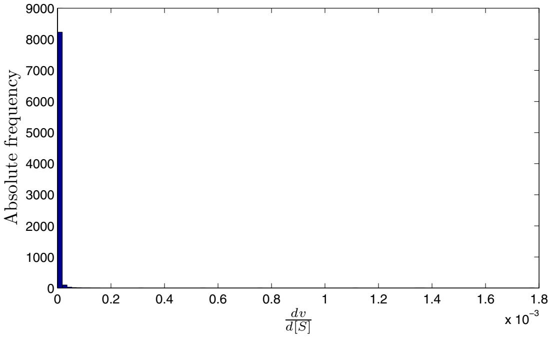

(c) Histogram of the BRENDA distribution.

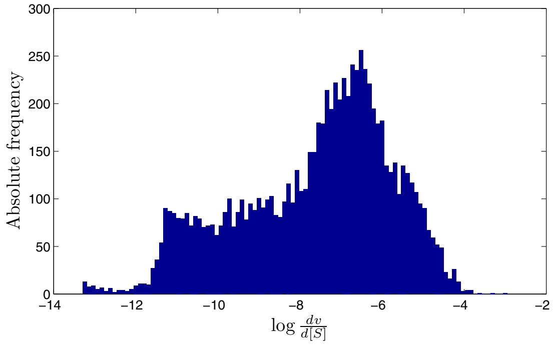

(d) Histogram of the $\log_2$-transformed BRENDA distribution.

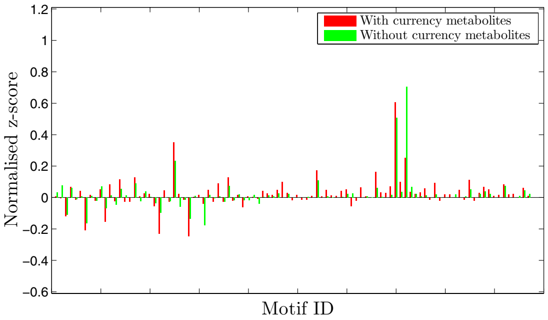

(e) NZSes of the full and reduced network.

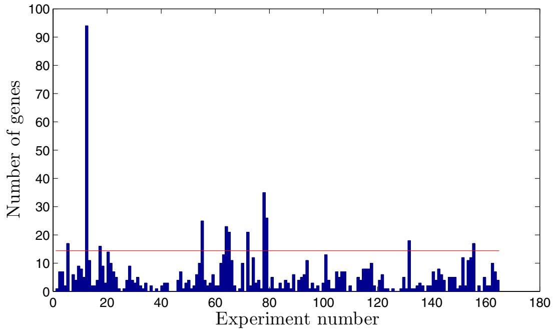

(f) NZSes of the full and reduced network.

Figure 14: **Figs. (a) and (b):** The motifs are sorted first according to density class, which is determined by the number of edges. Inside a density class the motifs are sorted by descending SSS. The red dashed lines divide the x-axes into 5 different density classes. The first class consists of motifs with 3 edges, the last class of the fully connected motif with 6 edges. See Fig. 9(a) for examples of the 13 motifs used here. The bar crossing the x-axis in [b] represents an NZS with infinite value, resulting from dividing by zero in(4).
Figs. (c) and (d): Histograms of $\frac{dv}{dx}$ and $ln(\frac{dv}{dx})$. In (c) we can see that there are many small values, $\pm 10^{-7}$ on average, and only a very few higher values (maximum: 1.1×10^{-3}). (d) shows the variation in the small values, which is quite large. The histograms of vectors $\boldsymbol{j}_{pos}$, $\boldsymbol{j}_{neg}$, $\boldsymbol{j}_{com}$ and $\boldsymbol{j}_{diag}$ are scaled and/or mirrored versions of the histogram in Fig. 14(c). So the BRENDA distribution consists of a large amount of small values with high variation and a few larger values.
Fig. (e): Comparison of NZSes for the full and the reduced model. A subtraction of these two scores is plotted in Fig. 7(a).
Fig. (f): Histograms of maximum gene expressions. A bar at position x with height y indicates that y genes had their maximal expression over all experiments in experiment x. The gene expression data source used here is RMA.

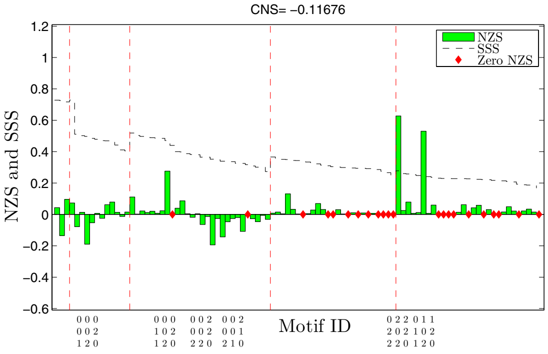

(a) RMA, $\tau = 0{,}05$. Average number of enzymes deleted: 45, average number of reactions deleted: 123.

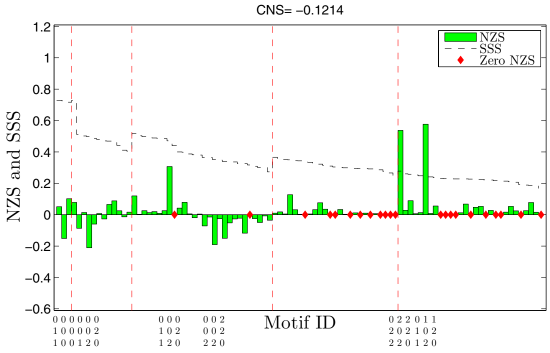

(b) RMA, $\tau = 0{,}1$. Average number of enzymes deleted: 78, average number of reactions deleted: 198.

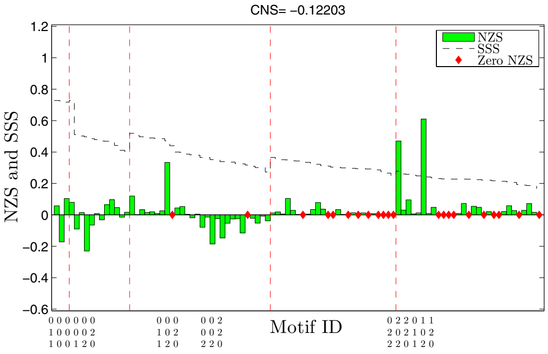

(c) RMA, $\tau = 0{,}2$. Average number of enzymes deleted: 141, average number of reactions deleted: 301.

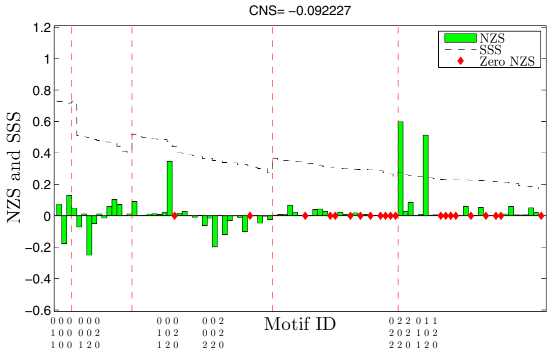

(d) RMA, $\tau = 0{,}4$. Average number of enzymes deleted: 342, average number of reactions deleted: 621.

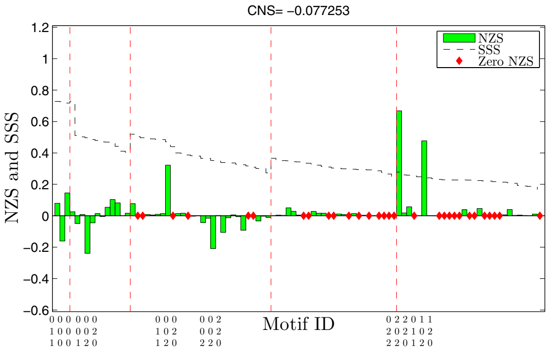

(e) RMA, $\tau = 0{,}5$. Average number of enzymes deleted: 481, average number of reactions deleted: 824.

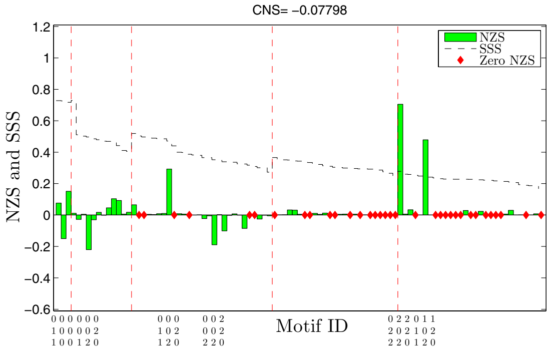

(f) RMA, $\tau = 0{,}6$. Average number of enzymes deleted: 617, average number of reactions deleted: 994.

Figure 15: RMA results for all six values for τ_1.

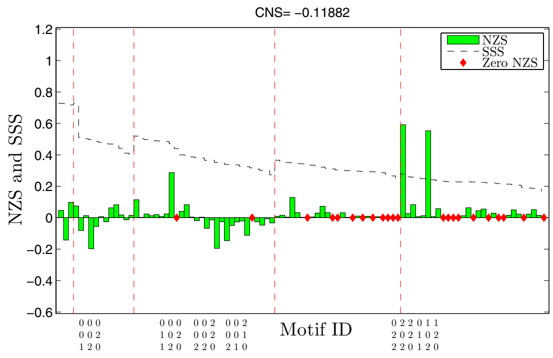

(a) MAS, $\tau = 0{,}05$. Average number of enzymes deleted: 58, average number of reactions deleted: 148.

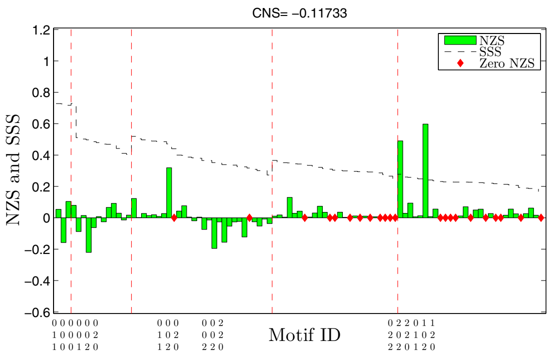

(b) MAS, $\tau = 0{,}1$. Average number of enzymes deleted: 95, average number of reactions deleted: 221.

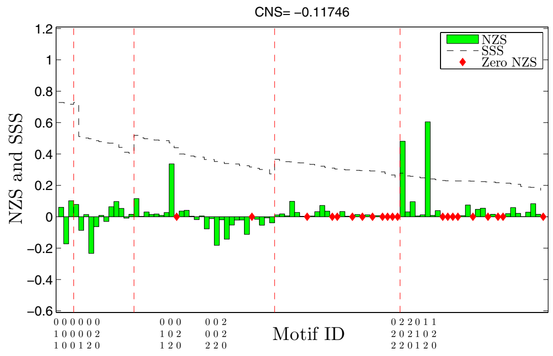

(c) MAS, $\tau = 0{,}2$. Average number of enzymes deleted: 168, average number of reactions deleted: 337.

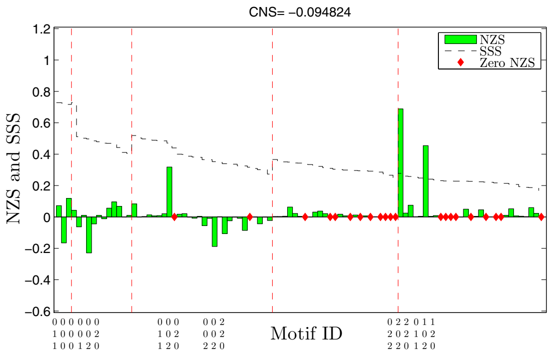

(d) MAS, $\tau = 0{,}4$. Average number of enzymes deleted: 390, average number of reactions deleted: 675.

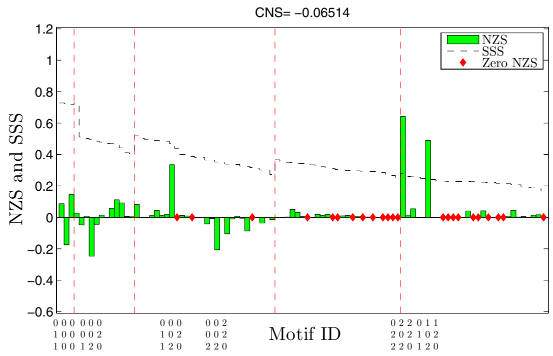

(e) MAS, $\tau = 0{,}5$. Average number of enzymes deleted: 527, average number of reactions deleted: 869.

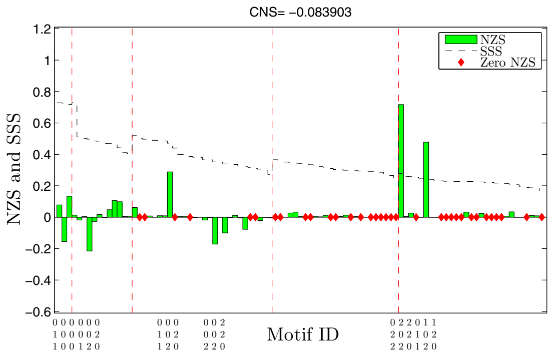

(f) MAS, $\tau = 0{,}6$. Average number of enzymes deleted: 652, average number of reactions deleted: 1024.

Figure 16: MAS results for all six values for τ_1.

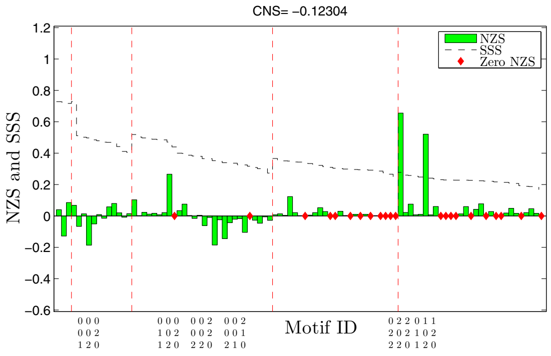

(a) P, $\tau = 10^{-1.5}$. Average number of enzymes deleted: 50, average number of reactions deleted: 100.

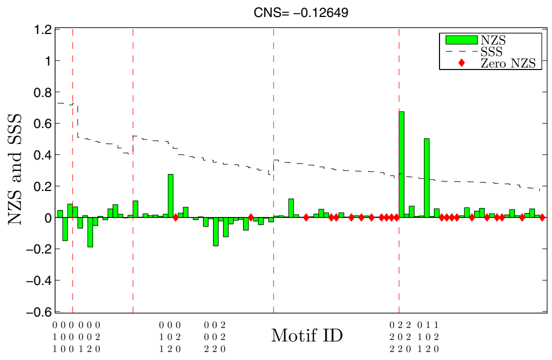

(b) P, $\tau = 10^{-2.5}$. Average number of enzymes deleted: 104, average number of reactions deleted: 198.

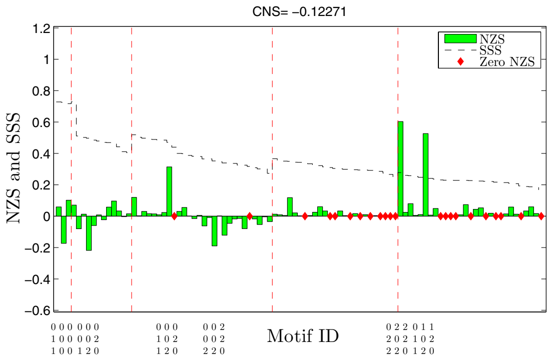

(c) P, $\tau = 10^{-3}$. Average number of enzymes deleted: 159, average number of reactions deleted: 293.

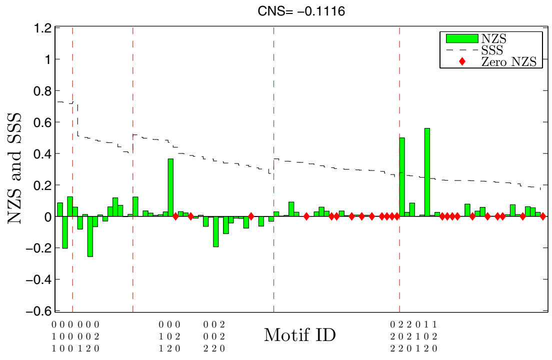

(d) P, $\tau = 10^{-3.5}$. Average number of enzymes deleted: 326, average number of reactions deleted: 552.

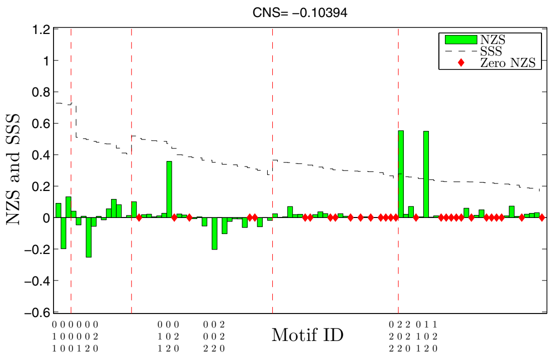

(e) P, $\tau = 10^{-3.6}$. Average number of enzymes deleted: 435, average number of reactions deleted: 713

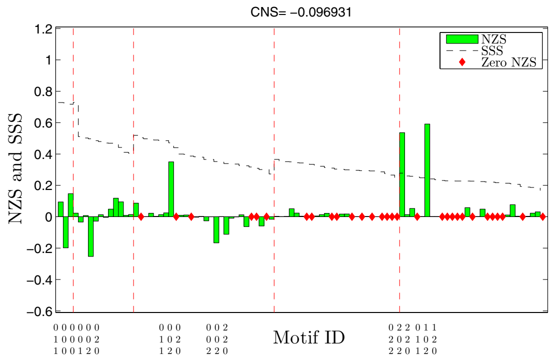

(f) P, $\tau = 10^{-3.65}$. Average number of enzymes deleted: 559, average number of reactions deleted: 887.

Figure 17: P-value results for all six values for τ_2.

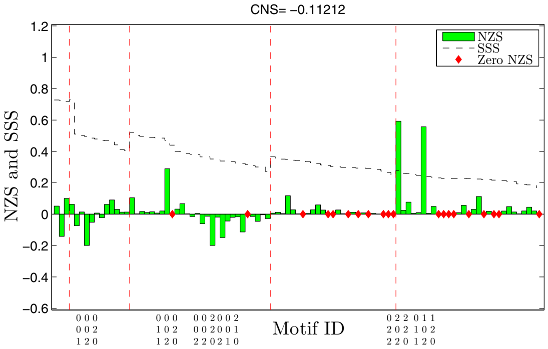

(a) Random, 70 enzymes randomly deleted.

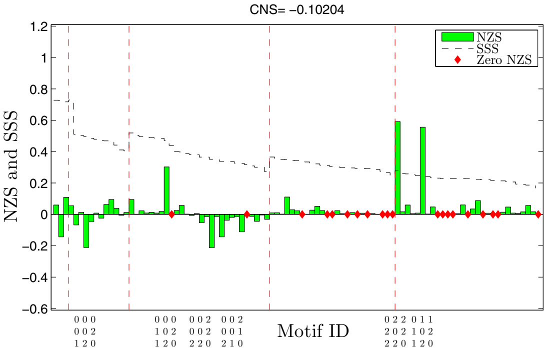

(b) Random, 120 enzymes randomly deleted.

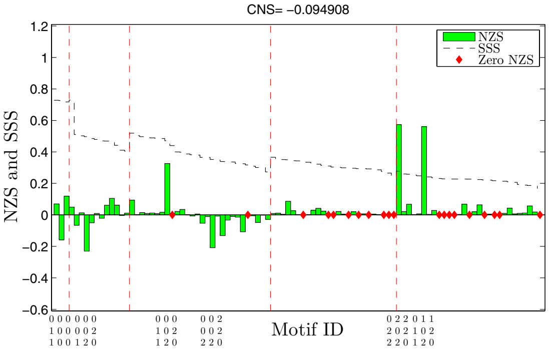

(c) Random, 200 enzymes randomly deleted.

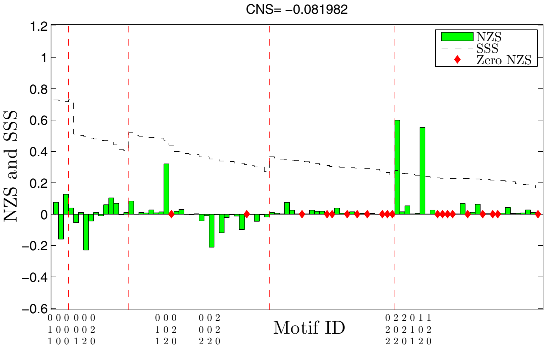

(d) Random, 400 enzymes randomly deleted.

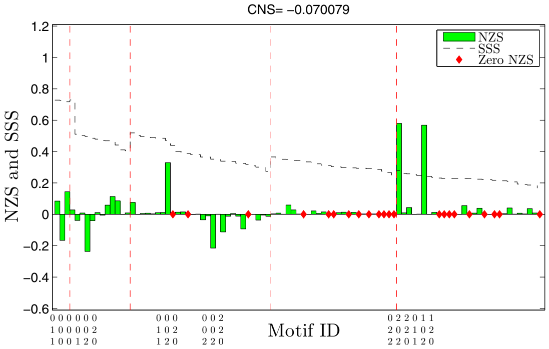

(e) Random, 500 enzymes randomly deleted.

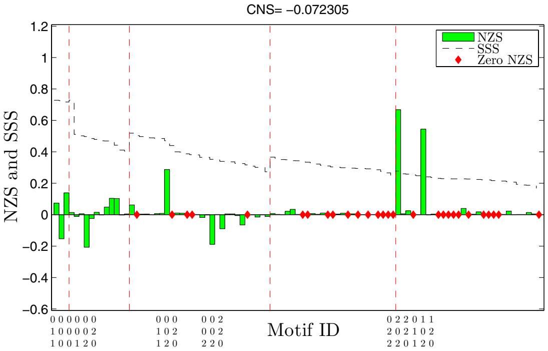

(f) Random, 600 enzymes randomly deleted.

Figure 18: Results for networks with randomly discarded reactions.

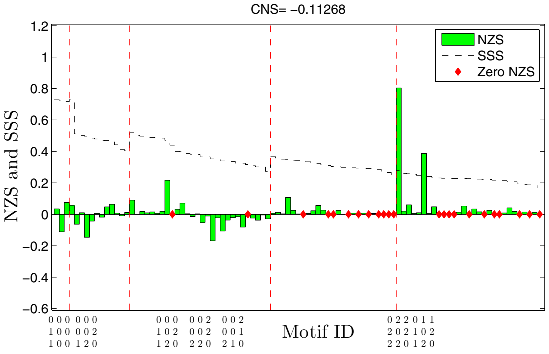

(a) RMA with $\tau_1 = 0.05$.

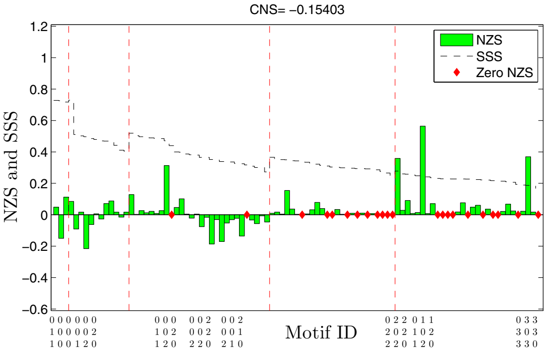

(b) RMA with $\tau = 0.05$.

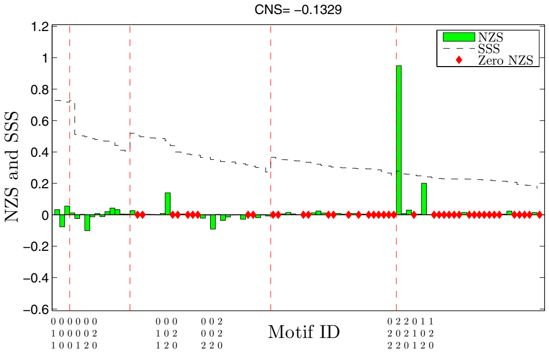

(c) RMA with $\tau = 0.4$.

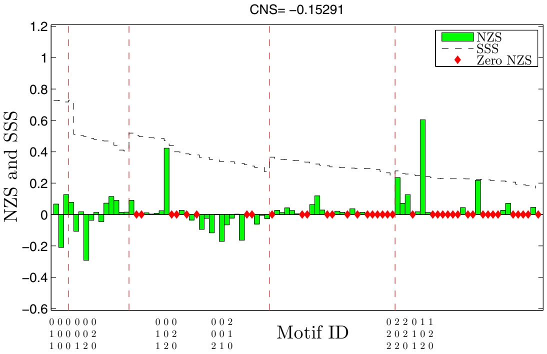

(d) RMA with $\tau = 0.4$.

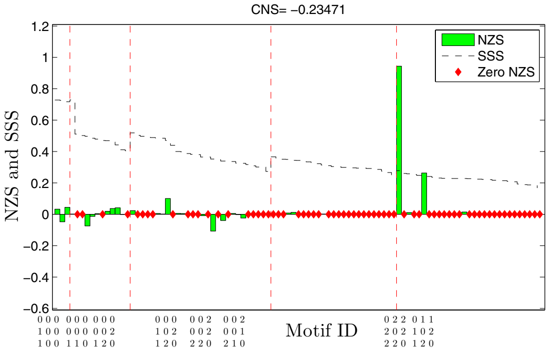

(e) Random removal of 600 genes.

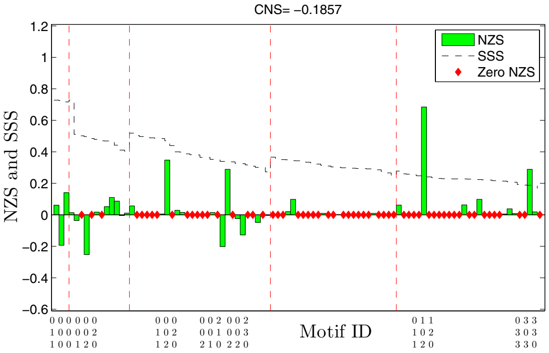

(f) Random removal of 600 genes.

Figure 19: Plots for a few chosen experiments. Each pair of plots ((a) and (b), (c) and (d), and (d) and (e)) are the two NZS profiles showing the lowest correlation within that experiment.

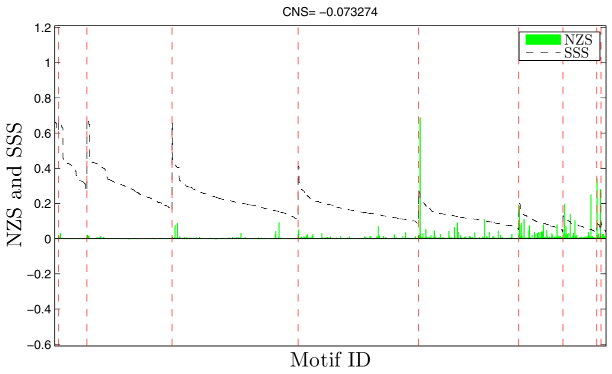

(a) NZS vs SSS 4

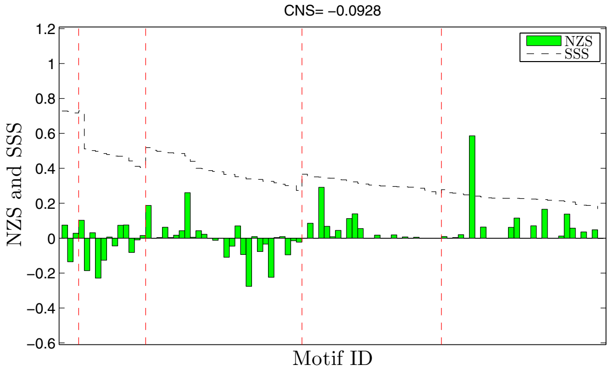

(b) E. Coli: NZS and Brenda SSS

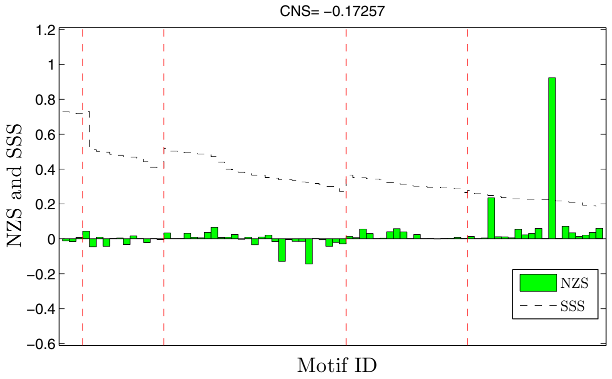

(c) H. Sapiens: NZS and Brenda SSS

Figure 20: (a) Results of method E from Table 1 using motifs of size 4. (b) Results of using method E in Table 1 on a metabolic network of *E. Coli*. (c) Results of using method E in Table 1 on a metabolic network of *H. Sapiens*.

9 Supplementary equations

The values for $[E]$ in [Ghaemmaghami et al., 2003] have units molecules per cell. In order to change the units to moles per liter, we use (14).

$$[E]_c = \frac{[E]}{\text{Cell volume} \cdot \text{Avogadro's number}} \tag{14}$$

with:

- Cell volume $= 29 \cdot 10^{-15}$ as in[1];
- Avogadro's number $= 6.0221 \cdot 10^{23}$.

[1] `http://yeastpheromonemodel.org/wiki/Cell_volume`
